# Substance use frequency and associations with chronic pain among a cohort of people who inject drugs in Montreal, Canada

**DOI:** 10.1080/24740527.2025.2598284

**Published:** 2026-02-04

**Authors:** Sasha Udhesister, Sarah Larney, M. Gabrielle Pagé, Nanor Minoyan, Stine Bordier Høj, Valérie Martel-Laferrière, Didier Jutras-Aswad, Julie Bruneau

**Affiliations:** a Centre de Recherche du Centre Hospitalier de l’Université de Montréal, Montréal, Canada; b Département de Médecine Familiale et Médecine d’Urgence, Université de Montréal, Montréal, Canada; c Département d’anesthésiologie et médecine de la douleur, Université de Montréal, Montréal, Canada; d Département de psychologie, Université de Montréal, Montréal, Canada; e Département de médecine sociale et préventive, École de Santé Publique de l’Université de Montréal, Montréal, Canada; f Département de Microbiologie, Infectiologie et Immunologie, Université de Montréal, Montréal, Canada; g Département de Psychiatrie et addictologie, Université de Montréal, Montréal, Canada

**Keywords:** Chronic pain, people who inject drugs, substance use, alcohol use

## Abstract

**Background:**

The relationship between substance use and chronic pain is bidirectional. Although chronic pain and polysubstance use are highly prevalent among people who inject drugs (PWID), few studies have examined how the frequency of use of different substances relates to chronic pain.

**Aims:**

The aim of this study was to examine associations between substance use frequency and chronic pain in a sample of PWID.

**Methods:**

A cross-sectional analysis was conducted among PWID participating in a community-based cohort in Montreal, Canada. Chronic pain measures were introduced in the interviewer-administered questionnaire in February 2017. The first questionnaire was used for analyses, which covers data up to November 2022. Logistic regression analyses were conducted to examine associations between alcohol, stimulants, opioid and cannabis frequency and chronic pain.

**Results:**

Six hundred and eight participants were included; 84% were men and mean age was 44.7 years old. Prevalence of chronic pain was 48%. Age (adjusted odds ratio [aOR] = 1.38, 95% confidence interval [CI] 1.15–1.65) and regular alcohol consumption in the past month (aOR = 1.76, 95% CI 1.13–2.75) were associated with chronic pain in univariable and multivariable logistic regression models. The frequency of use for all other substances was not found to be significantly associated with chronic pain.

**Conclusion:**

The prevalence of chronic pain in our sample was high. The positive association between high frequency of alcohol use and chronic pain could be explained by alterations of pain pathways by heavy use and withdrawal episodes, potentially increasing hyperalgesia. This study underscores the importance of addressing alcohol use along with other substances among PWID.

## RÉSUMÉ

**Contexte:** La relation entre la consommation de substances et la douleur chronique est bidirectionnelle.

Bien que la douleur chronique et la polyconsommation de substances soient très répandues chez les personnes qui s’injectent des drogues, peu d’études se sont penchées sur le lien entre la fréquence d’utilisation de différentes substances et la douleur chronique.

**Objectif:** Cette étude visait à examiner les associations entre la fréquence de consommation de substances et la douleur chronique dans un échantillon de personnes qui s’injectent des drogues.

**Méthodes:** Une analyse transversale a été réalisée auprès de personnes qui s’injectent des drogues participant à une cohorte communautaire à Montréal, au Canada. Des mesures de la douleur chronique ont été intégrées au questionnaire administré par un intervieweur en février 2017. Le premier questionnaire comprenant ces mesures a été utilisé pour les analyses, incluant des données jusqu’en novembre 2022. Des analyses de régression logistique ont été menées pour examiner les associations entre la fréquence de consommation d’alcool, de stimulants, d’opioïdes et de cannabis et la douleur chronique.

**Résultats:** Au total, 608 participants ont été inclus; 84 % étaient des hommes; l’âge moyen était de 44,7 ans. La prévalence de la douleur chronique était de 48 %. L’âge (rapport de cotes ajusté [RCA] = 1,38; intervalle de confiance à 95 % [IC] 1,15–1,65) et la consommation régulière d’alcool dans le mois précédent (RCA = 1,76; IC à 95 % 1,13–2,75) étaient associés à la douleur chronique dans les modèles de régression logistique univariés et multivariés. La fréquence d’utilisation de toutes les autres substances n’était pas significativement associée à la douleur chronique.

**Conclusion:** La prévalence de la douleur chronique dans notre échantillon était élevée. L’association positive entre la fréquence élevée de consommation d’alcool et la douleur chronique pourrait s’expliquer par des altérations des voies de la douleur attribuables à une consommation élevée et à des épisodes de sevrage, susceptibles d’accroître l’hyperalgésie. Cette étude souligne l’importance de s’attaquer à la consommation d’alcool, de même qu’à celle d’autres substances, chez les personnes qui s’injectent des drogues.

## Introduction

The prevalence of chronic pain, defined as intermittent or continuous pain lasting 3 months, ranges from 18% to 22% among adults worldwide.^1,2^ Substance use in relation to chronic pain has been widely studied, with increasing consensus about the concept of a reciprocal relationship between chronic pain and substance use.^3^ People report using substances—most frequently opioids, cannabis, and alcohol—to self-medicate pain symptoms, with studies showing mixed results or modest positive outcomes at best.^4–7^ Conversely, self-medication is a major driver of ongoing substance use, risk for substance use disorders, and the onset and severity of chronic pain.^3,8^ This bidirectionality is predominantly documented for alcohol. Although acute alcohol use can transiently reduce pain, chronic or heavy use and withdrawal are associated with neuroinflammatory changes and alterations in pain pathways, contributing to hyperalgesia.^3,9,10^ There are indications that prolonged opioid use and regular cannabis use can also lead to opioid-induced hyperalgesia and cannabis-induced hyperalgesia, respectively.^11–13^ In all cases, the regular use of these substances can lead to alterations in an individual’s pain tolerance and sensitivity, leading to chronic pain.^3,7,9,11–14^

Chronic pain is highly prevalent among people who use drugs, particularly those who inject drugs, with rates estimated to be at least twice as high as in the general population.^15,16^ Polysubstance users also show substantially higher adjusted odds of chronic pain compared to single-substance users or nonusers.^17^ Though opioids are the primary drug injected globally, Canadian data indicate that opioids (45.7%) and stimulants (46.5%) are reported at similar rates as the main drug injected.^18^ Cohort studies in Canada further highlight the high prevalence of both injection and noninjection polysubstance use, including alcohol and cannabis, among PWID.^19,20^

PWID represent a key population in understanding the association with polysubstance use and chronic pain, because injection practices, heavy use of multiple drugs, and related injuries are intertwined and can result in painful long-term complications.^21,22^ Among PWID, studies exploring chronic pain tend to focus on pain management strategies such as self-management of pain and illicit substance use.^8,15,23^ Studies that investigate substance use and chronic pain among PWID often aim to explore the nonmedical use of opioids, with few exploring medical opioid use, such as methadone prescribed for opioid agonist therapy (OAT). In a recent study of PWID recruited from a syringe service program in Baltimore, three-quarters reported using substances to manage chronic pain, and rates of nonmedical opioid use did not differ between those with and without pain.^15^ Another study conducted among PWID in San Francisco found that past 24-h nonmedical prescription opioid use was associated with increased levels of pain intensity and interference,^24^ supporting the concept of reciprocity. Also, a study of PWID in Vancouver found that there is no significant association between self-management of pain and methadone enrollment,^8^ yet there is scarce evidence that denial of prescription analgesia is significantly associated with methadone enrollment, as observed in another study conducted among PWID in Vancouver.^23^

Patterns of polysubstance use and their differential impacts on chronic pain outcomes are not well described. The frequency of substance use, including opioids, stimulants, cannabis, and most specifically alcohol, is of particular interest, because frequency of use is a specific pattern that provides insight into the regular use of substances and withdrawal avoidance, both of which have been observed to be key components in the substance-induced chronic pain pathway.^3^ Exploring these relations could provide information for tailoring messages and interventions in addiction care settings. The objectives of this study are to examine the relations between frequency of substance use and chronic pain in a population of community-based recruited PWID.

## Methods

### Study design and setting

This study used a cross-sectional sample obtained from the HEPatitis COhort (HEPCO), an ongoing prospective community-based cohort study of PWID recruited and followed in Montreal. Participants are recruited through street-level strategies and community-based organizations. To be eligible for inclusion in the cohort, individuals must be 18 years or older, report injection drug use in the past 6 months, speak English or French, and reside in the Greater Montreal Area.

The cohort was established in 2004 to examine the determinants and incidence of hepatitis C virus (HCV) infection in this population and has undergone several revisions to incorporate emerging subject areas of interest. The HEPCO questionnaire includes detailed sociodemographic, drug use, behavioral, and health service utilization data using a structured questionnaire. Interviewer-administered questionnaires were used to collect data at each visit. Briefly, follow-up visits are conducted every 3 months with venous blood sampling performed for HCV antibody and RNA testing for incidence-related objectives. Details on HEPCO recruitment and follow-up have been previously described.^25,26^ In February 2017, specific measures on chronic pain and related questions were introduced in the questionnaire and administered to all participants.

### Analytic sample

Data from the first study visit by participants between February 2017 and November 2022 were included in this analysis, corresponding to the introduction of chronic pain measures. Participants reporting cancer as the reason for their pain were excluded (*n* = 2), given our focus on chronic noncancer pain.

### Ethics

Ethical approval was provided by the Comité d’éthique de la recherche du Center hospitalier de l’Université de Montréal (CHUM Research Ethics Board Approval No. 17.096). All participants provided written informed consent and received CA$20 to CA$40 at each visit as compensation for their time.

### Measures

#### Chronic pain

The presence of chronic pain was defined as pain lasting more than 3 months (yes/no), using the following question taken from the Brief Pain Inventory^27^:

“throughout our life, most of us have pain from time to time (headache, toothache). Except for these kinds of pain, are you currently experiencing chronic pain, that is to say, pain that has been present for three months or more (e.g., persistent back pain, arthritis, etc.)”.

#### Substance use

Data included the injection and noninjection use of substances (yes/no) in the past month: heroin, nonmedical pharmaceutical opioids, cocaine, amphetamines, tranquilizers (i.e., benzodiazepines), cannabis, and alcohol. The number of days of use for each substance in the past month was also reported. For alcohol, regular use was defined as consuming alcohol every other day or more in the past month (i.e., 15 or more days), as a threshold likely to represent high risk of withdrawal, and occasional use was any use less than this (i.e., 1–14 days).^28^ For other substances, frequency was categorized into three levels: none, occasional, and regular use, based on definitions determined previously from work on our cohort,^28,29^ the U.S. Office of National Drug Control Policy,^30^ and also corresponding to categories used recently in a similar cohort in Vancouver.^31^ For heroin, nonmedical pharmaceutical opioid use, cocaine, amphetamines, tranquilizers, and cannabis, occasional use was defined as use for 1 to 3 days, and regular use was defined as use on four or more days in the past month, indicative of average use less than once a week and use at least once a week per month, respectively.^28–30^ On August 14, 2017, while the study was ongoing, an additional question was added to the baseline questionnaire asking whether participants had used cannabis prescribed to them in the past 3 months. Two hundred and twenty-two participants who had not yet answered the baseline questionnaire prior to this addition responded to this question in the updated baseline questionnaire.

#### Covariates

Covariate selection was informed by the literature based on clinical relevance and relevance to experiences of PWID. These included age,^15,16,24^ sex,^16,32^ and OAT enrollment^33,34^ in the past 3 months.

#### Chronic pain characteristics

Participants reporting chronic pain were administered the chronic pain questionnaire module, which included a modified version of the Brief Pain Inventory to capture data on pain intensity (average in the past month on a numerical rating scale: 0 = no pain, 10 = the worst pain possible) and pain interference (interference with ten aspects of daily function in the past month on a numerical rating scale: 0 = no interference, 10 = complete interference).^35^ The Brief Pain Inventory has been validated in several populations and for use in different languages.^16,24^ It has also been used in many studies investigating chronic pain among people with substance use disorders, including PWID.^16,24,36^ The original Brief Pain Inventory includes seven aspects of daily functioning to be measured for pain interference (interference with general activity, mood, walking ability, normal work [including housework], relations with others, sleep, and enjoyment of life).^27^ This version of the Brief Pain Inventory was modified and has been validated in other studies and allows us to obtain a broader sample of areas that could potentially be affected by pain, including three additional aspects of daily functioning (personal care, recreational activities, and social activities).^35^ In accordance with the Brief Pain Inventory User Guide, a measure of average pain interference was obtained by determining the average score of the ten daily functions measured using the Brief Pain Inventory.^27,36^ In addition to the Brief Pain Inventory, participants were asked questions on pain characteristics (circumstance[s] surrounding pain onset, site, age at pain onset, duration of pain, having continuous pain in the past month [yes/no]) and a question on medication use for pain in the past 3 months (yes/no).

### Statistical analysis

Descriptive statistics were used to characterize the sample and specifically chronic pain in terms of the circumstance(s) surrounding pain onset, site, age of pain onset, duration, presence of continuous pain, intensity, interference, and medication use for chronic pain. Univariable and multivariable logistic regression models were used to identify the associations between substance use frequency and presence of chronic pain. Variables included in the univariable analysis included age (continuous and reported as 10-year increments), sex, the number of days of use of various substances (none, occasional, or regular use), and OAT enrollment. Two models were created for the multivariable analysis. Model 1 included all substance use and sociodemographic variables. Purposeful selection was used to determine covariates for model 2.^37,38^ This method allows for the inclusion of significant covariates and important confounding variables based on statistical significance and clinical relevance. Variables that had a *P* value <0.25 from the univariable analysis were primarily included in the multivariable analysis, and, following an iterative process, any covariates that were not found to be statistically significant (i.e., *P* < 0.10) or were not observed to act as confounders (i.e., estimates for other variables did not change by >15% when removing the variable in question) were removed from the final model.^37,38^ During the multivariable analysis, we observed that including age in the model resulted in a drastic shift in the association of OAT, which was not observed for any other variables. To explore this further, we conducted a post hoc analysis, stratifying the sample by age (median split) to observe the association between OAT and chronic pain in younger and older participants. Odds ratios and 95% confidence intervals are reported. Analyses were performed using R Statistical Software (v4.1.2).^39^

## Results

### Study sample

Among 608 participants, the median age was 44.7 years (interquartile range [IQR] = 36.3–52.8), and 83.9% were men (Table 1). A quarter of the sample reported heroin use (25.7%; 96% injected), 34.5% reported nonmedical pharmaceutical opioid use (90% injected), and over half reported alcohol use in the past month (54.4%). Forty percent of participants were on OAT. Just 7.2% of participants reported using cannabis prescribed to them in the past 3 months (16/222).

**Table 1.** Univariable and multivariable associations with chronic pain among PWID (*n* = 608).

Variable	Chronic pain, *n* (%) or median (IQR)	No Chronic pain, *n* (%) or median (IQR)	Univariable, OR (CI)	Model 1: aOR (CI)	Model 2: aOR (CI)
**Age** (10-year increments)	46.6 (38–54)	42.9 (35–51)	1.32 (1.13–1.54)	1.38 (1.15–1.65)	1.34 (1.14–1.59)
**Sex**					
Female	39 (13.4)	59 (18.7)	Ref	Ref	Ref
Male	253 (86.6)	257 (81.3)	1.49 (0.96–2.33)	1.26 (0.79–2.02)	1.30 (0.83–2.07)
**Alcohol use^a^**					
None	118 (40.4)	159 (50.3)	Ref	Ref	Ref
Occasional	99 (33.9)	97 (30.7)	1.38 (0.95–1.99)	1.42 (0.96–2.10)	1.40 (0.96–2.05)
Regular	75 (25.7)	60 (19.0)	1.68 (1.11–2.56)	1.76 (1.13–2.75)	1.70 (1.11–2.61)
**Heroin use^a^**					
None	222 (76.0)	230 (72.8)	Ref	Ref	
Occasional	29 (10.0)	30 (9.5)	1.00 (0.58–1.73)	0.96 (0.52–1.76)	
Regular	41 (14.0)	56 (17.7)	0.76 (0.48–1.18)	0.83 (0.51–1.36)	
**Nonmedical pharmaceutical opioid use^a^**					
None	195 (66.8)	203 (64.2)	Ref	Ref	
Occasional	21 (7.2)	28 (8.9)	0.78 (0.42–1.42)	0.91 (0.47–1.75)	
Regular	76 (26.0)	85 (26.9)	0.93 (0.64–1.34)	1.18 (0.77–1.80)	
**Cocaine use^a^**					
None	124 (42.5)	123 (38.9)	Ref	Ref	
Occasional	48 (16.4)	53 (16.8)	0.90 (0.56–1.43)	0.93 (0.57–1.53)	
Regular	120 (41.1)	140 (44.3)	0.85 (0.60–1.20)	0.79 (0.54–1.16)	
**Tranquilizer use^a^**					
None	255 (87.3)	281 (88.9)	Ref	Ref	
Occasional	17 (5.8)	15 (4.7)	1.25 (0.61–2.58)	1.19 (0.55–2.62)	
Regular	20 (6.8)	20 (6.3)	1.10 (0.58–2.10)	0.94 (0.46–1.90)	
**Amphetamine use**^a^					
None	209 (71.8)	226 (71.5)	Ref	Ref	
Occasional	32 (11.0)	39 (12.3)	0.89 (0.53–1.47)	0.96 (0.55–1.66)	
Regular	50 (17.2)	51 (16.1)	1.06 (0.69–1.64)	1.17 (0.73–1.88)	
**Cannabis use^a^**					
None	124 (42.5)	143 (45.3)	Ref	Ref	
Occasional	30 (10.3)	40 (12.7)	0.86 (0.51–1.47)	0.82 (0.47–1.44)	
Regular	138 (47.3)	133 (42.1)	1.20 (0.85–1.68)	1.18 (0.82–1.70)	
**OAT^b^**					
Yes	120 (41.2)	122 (38.9)	1.10 (0.80–1.53)	**1.49 (1.03–2.18)**	1.41 (1.00–2.00)
No	171 (58.8)	192 (61.1)	Ref	Ref	REF

^a^Past month; ^b^Past three months; OR= Odds ratio; aOR= adjusted odds ratio; CI= 95% confidence interval. Occasional and regular use for alcohol: 1–14 days and 15 or more days in the past month, respectively; Occasional and regular use for heroin, nonmedical pharmaceutical opioids, cocaine, amphetamines, tranquilizers, cannabis: 1–3 days and four or more days in the past month, respectively.

Of the 292 participants who reported having chronic pain (48.0%), the most common reasons for chronic pain were accidents (35.6%; including motorized vehicle accidents, workplace accidents, sports accidents, accidents at home, and accidents in a public space), followed by no specific cause (19.5%; Table 2). The mean age of onset of chronic pain was 32.4 years (SD 13.6), with more than half the sample reporting living with chronic pain for more than 10 years (51.7%). The average pain intensity and pain interference reported in the past month were moderate (5.79 and 4.16 out of 10, respectively).^16,27^ Less than half of the participants reported using prescribed pain medication in the past 3 months (45.7%; 133/291).

**Table 2.** Chronic pain characterization among a sample of PWID reporting chronic pain (n = 292).

Pain measures	Category	Findings n (%) or Mean (±SD)	
Circumstance(s) surrounding pain onset, *n* (%)	Accident		104 (35.6)
	Nothing specific	57 (19.5)	
	Medical condition	38 (13.0)	
	Repetitive movement/trauma	30 (10.3)	
	Fight/assault	28 (9.6)	
	Other	54 (18.5)	
Site of pain, *n* (%)^a^	Lower limbs	85 (29.5)	
	Lower back	68 (23.6)	
	Upper limbs	29 (10.1)	
	Shoulder	23 (8.0)	
	Head and neck	22 (7.6)	
	Chest and abdomen	20 (6.9)	
	Other	41 (14.2)	
Duration of pain, *n* (%)	Less than 1 year	24 (8.2)	
	≥1 year <5 years	66 (22.6)	
	≥5 years <10 years	51 (17.5)	
	10+ years	151 (51.7)	
Reporting continuous pain, *n* (%)^b^	Yes	227 (77.7)	
Average pain intensity, mean (SD)^b^		5.79 (2.28)	
Average pain interference, mean (SD)^b^		4.16 (2.40)	

^a^Four participants did not reply. ^b^Past month.

### Correlates of chronic pain

Table 1 presents the characteristics of participants according to chronic pain and the univariable and multivariable associations with the presence of chronic pain within the sample. In univariable analysis, age (10-year increments: odds ratio [OR] = 1.32, 95% confidence interval [CI] 1.13–1.54) and regular alcohol consumption in the past month (OR = 1.68, 95% CI 1.11–2.56) were independently associated with chronic pain. In adjusted models 1 and 2, age (10-year increments: model 1 adjusted odds ratio [aOR] = 1.38, 95% CI 1.15–1.65; model 2 aOR = 1.34, 95% CI 1.14–1.59) and regular alcohol consumption in the past month (model 1 aOR = 1.76, 95% CI 1.13–2.75; model 2 aOR = 1.70, 95% CI 1.11–2.61) were positively associated with chronic pain. In adjusted model 1, OAT (aOR = 1.49 95% CI 1.03–2.18) was also positively associated with chronic pain. No other substance or sociodemographic factor estimates were observed to exclude the null value. In sensitivity analyses, results were consistent when regular use of all substances was defined as more than 15 days per month, as for alcohol use (see supplemental material). Following stratification by age (dichotomized at median age of 44.7 years), in post hoc analysis, for the association between OAT and chronic pain, OAT was positively associated with chronic pain among the older participants (OR = 1.66, 95% CI 1.01–2.77), whereas there was no evidence of an association among the younger participants (OR = 0.95, 95% CI 0.60–1.50).

## Discussion

Similar to other studies among PWID, almost half of this sample reported having chronic pain, which is more than double that of the general population^40^ and as high as that among other high-risk populations such as military veterans.^41^ For many participants, chronic pain was long-standing and continuous. Older age and regular alcohol use (every other day or more) in the past month were significantly associated with chronic pain in both univariable and multivariable analyses. No other substance was associated with chronic pain in this population. We also found that current OAT exposure was associated with increased odds of having chronic pain among participants who were over 44.7 years of age but not among the younger ones.

Regular alcohol consumption was significantly associated with chronic pain, supporting the hypothesis that in this population, regular alcohol use may exacerbate pain through withdrawal-related symptoms and alcohol-induced hyperalgesia. There is evidence that alcohol use can lead to hyperalgesia, as in the case of alcohol-induced neuropathy, which is common among frequent heavy drinkers and also during alcohol withdrawal.^3,9^ Long-term excessive alcohol consumption has been associated with increased pain severity and the exacerbation of chronic pain through alterations in neural pathways and reduced pain thresholds.^14,42^ Our findings suggest that this mechanism may also operate among chronic polysubstance PWID, including those using opioids and stimulants, and even after taking into account regular use of these other substances, regular alcohol use was the only substance found to be significantly associated with chronic pain.

Although the frequency of use for other substances was not found to be associated with chronic pain, it is plausible that chronic exposure to opioids and possibly other substances has contributed at some point to the emergence of hyperalgesia but may not be currently related to pain exacerbation. There is qualitative literature noting that one of the main goals of using illicit substances for many chronic people who use drugs is to avoid withdrawal.^43^ It is possible that this drug use pattern does not trigger additional alteration of the neural pathways by which hyperalgesia is exacerbated. These questions would have to be further explored by examining concurrent combinations of drugs in relation to chronic pain prospectively.

Chronic pain was associated with older age in our study. Chronic pain is a comorbidity experienced more commonly by older adults and can have debilitating consequences on quality of life.^44^ Chronic pain may be attributed to a host of factors among older individuals in this population, such as increased comorbidities, changes in pain perception, and prolonged exposure to common painful experiences (i.e., violence and injection complications), which may pose additional barriers and a need for alternative measures to manage pain.^45,46^ Being on OAT was associated with chronic pain only among older participants in our sample. Oftentimes, OAT is prescribed for the purposes of managing opioid use disorders and co-occurring chronic pain^47^ and has been demonstrated to help improve pain outcomes.^48^ However, stigma and competing priorities can influence OAT access. A qualitative study from Montreal showed that people who use drugs and have chronic pain may be hesitant to initiate OAT to manage their pain, because managing their substance use is not their objective, and many believe that this recommendation is driven by physicians’ underlying desire to manage substance use and not chronic pain.^49^

Because chronic pain prevalence increases with age,^44,50^ older PWID may face growing challenges in self-medicating with illicit substances due to age- and pain-related limitations (i.e., difficulty obtaining money or accessing drugs).^43^ Consequently, aging PWID with chronic pain may become more inclined to use OAT as a means of pain management. Future studies could investigate factors facilitating pain management among PWID on OAT treatment according to their age and drug use trajectories. The lack of association among younger participants could indicate a lower average intensity and perhaps less perceived need for institutional pain management.^44,50^ Current literature indicates that chronic pain is particularly difficult to manage among PWID.^8^ Physicians are often reluctant to prescribe pain medications to this population due to concerns about tolerance, drug interactions, and increased risks of overdose.^8,51^ The management of co-occurring chronic pain and regular alcohol use introduces additional clinical and social barriers to effective treatment. These barriers are compounded by the perception that PWID are at risk for diversion, misuse, and dependence, further reinforcing physician caution and contributing to persistent gaps in care.^8,51^

Our findings suggest that regular alcohol use adversely affects chronic pain among PWID, consistent with evidence from the general population.^7,14^ Given that over half of the participants reported recent alcohol use, adapting existing pain screening and management strategies for individuals with alcohol use to the context of PWID is warranted. Further research is needed to guide the development of such interventions.

Accidents were the most reported circumstance surrounding chronic pain onset, which aligns with findings from clinical samples of individuals on OAT.^34,52^ A substantial portion of the sample also reported that the onset of their pain had no specific circumstances. This may be explained by social and environmental circumstances commonly experienced by PWID, which exacerbate and extend the duration of pain, including homelessness, exposure to violence, poor sleep quality, and physical comorbidities.^8,17,49^ A better understanding of the experiences and risks faced by PWID in relation to chronic pain genesis and persistence could present avenues for chronic pain prevention.

It would be worth considering the co-localization of services frequented by PWID, such as needle syringe programs, supervised consumption sites, or OAT services, with pain clinics or wound care services that could provide screening for chronic pain and evaluate risks related to chronic pain genesis. This may be particularly helpful for PWID who report regular alcohol consumption and may help with finding strategies to prevent chronic pain in this group or even slowing its progression. There is evidence suggesting that HIV/HCV treatment and service uptake among PWID are improved through co-localization with other types of services, such as needle syringe programs or OAT programs.^53–55^ Although the Canadian Action Plan for Pain has suggested more models of care that integrate pain services with harm reduction and substance use treatment services, these models of care are yet to be explored and should be considered in future research.^56^ Similarly, co-localization with health and social services provided by trusted community members (e.g., peers with lived experience, individuals who have a good rapport with the PWID community or have been trained to work specifically with this population) may lessen experiences of stigma and contribute to improved screening for chronic pain and pain outcomes among PWID.^53,54^

### Study limitations

This study used data collected from a long-standing, well-established cohort in Montreal and allowed for the exploration of the associations between substance use frequency and chronic pain among an aging population of PWID. The study is predisposed to the limitations of using data collected from a convenience sample, making it difficult to determine representativeness. Data are self-reported and therefore subject to bias from imperfect recall and social desirability bias, including underreporting or overreporting chronic pain.^15,49^ However, a recent review of self-report and illicit substance use found that self-report is a good measure of illicit drug use,^57^ and HEPCO interviewers are well trained on eliciting accurate information from participants. This exploratory study did not assess varying levels of pain intensity in relation to substance use frequency. The cross-sectional analysis precludes causal inferences from being made based on these findings but proposes avenues for future research.

## Conclusion

This study found that regular alcohol use was associated with chronic pain, whereas the regular use of other substances was not. Further investigation into chronic pain and various substance use patterns is still needed to drive insights into how substance use relates to chronic pain among PWID. Interventions are still needed for pain prevention and management among PWID, including those who use alcohol and other substances. Models of care consisting of co-localization of pain services (e.g., pain clinics) with other services frequented by PWID may also improve chronic pain screening and management in this population.

## Supplementary Material

Final Appendix Table_Udhesister Sept 29th.docx
