# Supplementary material for: Substance use frequency and associations with chronic pain among a cohort of people who inject drugs in Montreal, Canada
Source: Can J Pain. 2026 Feb 4;10(1):2598284. doi: 10.1080/24740527.2025.2598284 (PMC12885412; doi:10.1080/24740527.2025.2598284)
Supplement: Final Appendix Table_Udhesister Sept 29th.docx [file UCJP_A_2598284_SM7142.docx]

**APPENDIX**

**Table A1. Univariable and multivariable associations with chronic pain among PWID using alternative* definitions for substance use frequency categories (n=608)**

*****Use for alcohol, heroin, nonmedical pharmaceutical opioids, cocaine, amphetamines, tranquilizers, cannabis:

Occasional: 1-14 days

Regular: 15 or more days in the past month

| Variable | Chronic pain  n (%) or median (IQR) | No Chronic pain  n (%) or median (IQR) | Univariable  OR (CI) | Model 1:  aOR (CI) | Model 2:  aOR (CI) |
| --- | --- | --- | --- | --- | --- |
| Age (10-year increments) | 46.6 (38-54) | 42.9 (35-51) | **1.32 (1.13-1.54)** | **1.37 (1.15-1.64)** | **1.34 (1.14-1.59)** |
| Sex |  |  |  |  |  |
| Female | 39 (13.4) | 59 (18.7) | REF | REF | REF |
| Male | 253 (86.6) | 257 (81.3) | 1.49 (0.96-2.33) | 1.25 (0.78-2.00) | 1.30 (0.83-2.07) |
| Alcohol Use^1^ |  |  |  |  |  |
| None | 118 (40.4) | 159 (50.3) | REF | REF | REF |
| Occasional | 99 (33.9) | 97 (30.7) | 1.38 (0.95-1.99) | 1.41 (0.96-2.09) | 1.40 (0.96-2.05) |
| Regular | 75 (25.7) | 60 (19.0) | **1.68 (1.11-2.56)** | **1.74 (1.12-2.70)** | **1.70 (1.11-2.61)** |
| Heroin Use^1^ |  |  |  |  |  |
| None | 222 (76.0) | 230 (72.8) | REF | REF |  |
| Occasional | 49 (16.8) | 58 (18.4) | 0.88 (0.57-1.33) | 0.88 (0.54-1.42) |  |
| Regular | 21 (7.2) | 28 (8.9) | 0.78 (0.42-1.40) | 0.85 (0.45-1.61) |  |
| Nonmedical Pharmaceutical Opioid Use^1^ |  |  |  |  |  |
| None | 195 (66.8) | 203 (64.2) | REF | REF |  |
| Occasional | 47 (16.1) | 59 (18.7) | 0.83 (0.54-1.27) | 1.01 (0.63-1.63) |  |
| Regular | 50 (17.1) | 54 (17.1) | 0.96 (0.62-1.48) | 1.26 (0.77-2.08) |  |
| Cocaine Use^1^ |  |  |  |  |  |
| None | 124 (42.5) | 123 (38.9) | REF | REF |  |
| Occasional | 106 (36.3) | 119 (37.7) | 0.88 (0.62-1.27) | 0.82 (0.55-1.20) |  |
| Regular | 62 (21.2) | 74 (23.4) | 0.83 (0.55-1.26) | 0.78 (0.49-1.22) |  |
| Tranquilizer Use^1^ |  |  |  |  |  |
| None | 255 (87.3) | 281 (88.9) | REF | REF |  |
| Occasional | 29 (9.9) | 25 (7.9) | 1.28 (0.73-2.25) | 1.14 (0.62-2.13) |  |
| Regular | 8 (2.7) | 10 (3.2) | 0.88 (0.33-2.27) | 0.87 (0.31-2.34) |  |
| Amphetamine Use^1^ |  |  |  |  |  |
| None | 209 (71.8) | 226 (71.5) | REF | REF |  |
| Occasional | 59 (20.3) | 63 (19.9) | 1.01 (0.68-1.51) | 1.10 (0.71-1.72) |  |
| Regular | 23 (7.9) | 27 (8.5) | 0.92 (0.51-1.66) | 1.02 (0.53-1.94) |  |
| Cannabis use^1^ |  |  |  |  |  |
| None | 124 (42.5) | 143 (45.3) | REF | REF |  |
| Occasional | 72 (24.7) | 78 (24.7) | 1.06 (0.71-1.59) | 1.02 (0.66-1.56) |  |
| Regular | 96 (32.9) | 95 (30.1) | 1.17 (0.80-1.69) | 1.14 (0.77-1.70) |  |
| OAT^2^ |  |  |  |  |  |
| Yes | 120 (41.2) | 122 (38.9) | 1.10 (0.80-1.53) | 1.44 (0.99-2.09) | 1.41 (1.00-2.00) |
| No | 171 (58.8) | 192 (61.1) | REF | REF | REF |

^1^ Past month; ^2^ Past three months; OR= Odds ratio; aOR= adjusted odds ratio; CI= 95% confidence interval
